# Functionalized nanostructures for enhanced photocatalytic performance under solar light

**DOI:** 10.3762/bjnano.5.113

**Published:** 2014-07-09

**Authors:** Liejin Guo, Dengwei Jing, Maochang Liu, Yubin Chen, Shaohua Shen, Jinwen Shi, Kai Zhang

**Affiliations:** 1 International Research Center for Renewable Energy & State Key Laboratory of Multiphase Flow in Power Engineering, Xi’an Jiaotong University Xi’an 710049, China

**Keywords:** functionalized nanostructures, hydrogen, photocatalysis, photocatalytic, solar light

## Abstract

Photocatalytic hydrogen production from water has been considered to be one of the most promising solar-to-hydrogen conversion technologies. In the last decade, various functionalized nanostructures were designed to address the primary requirements for an efficient photocatalytic generation of hydrogen by using solar energy: visible-light activity, chemical stability, appropriate band-edge characteristics, and potential for low-cost fabrication. Our aim is to present a short review of our recent attempts that center on the above requirements. We begin with a brief introduction of photocatalysts coupling two or more semiconductors, followed by a further discussion of the heterostructures with improved matching of both band structures and crystal lattices. We then elaborate on the heterostructure design of the targeted materials from macroscopic regulation of compositions and phases, to the more precise control at the nanoscale, i.e., materials with the same compositions but different phases with certain band alignment. We conclude this review with perspectives on nanostructure design that might direct future research of this technology.

## Review

### Introduction

The increasing energy demand as well as the serious environmental contamination caused by the usage of fossil fuels give rise to the necessity to develop clean alternative fuels. Hydrogen, as a pollution-free and storable energy fuel, is a promising substitute of fossil fuels. Nowadays hydrogen is mainly manufactured from hydrocarbons such as fossil fuels, which limits its wide utilization. Therefore, the ability to economically and efficiently harvest hydrogen from renewable energies is central to advances in many areas and should be the fundamental research issue [1–4]. Photocatalytic hydrogen production from water by using solar energy is one of the most acceptable routes for this aim, since only abundant water and solar energy are needed for hydrogen production in the process. If the economic viability for industrial application is successfully satisfied, it will ultimately solve the energy and environmental problems [5–6].

**Figure 1:**
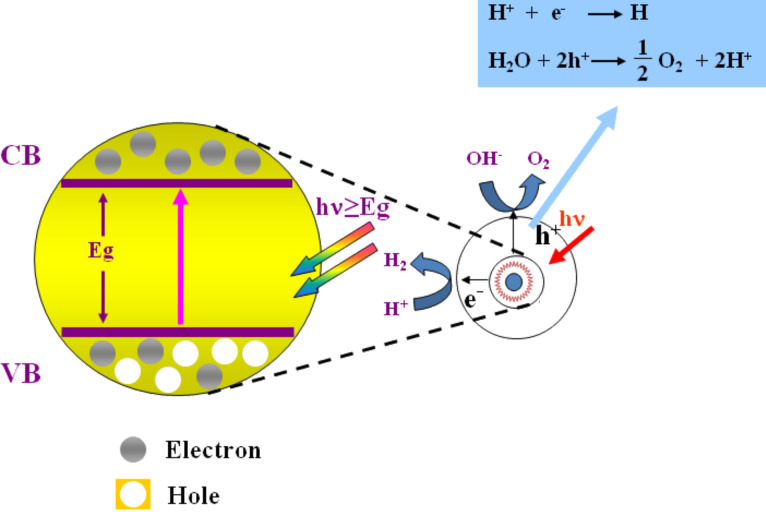
Basic principle for overall water splitting over semiconductor photocatalysts. Reprinted with permission from [6]. Copyright (2010) Elsevier.

Since the first report by Fujishima and Honda in 1972 [7], hydrogen production from water over semiconducting powders or films by using solar energy has been extensively studied. Thermodynamically, the reaction of producing hydrogen and oxygen from water splitting has a standard Gibbs free energy (Δ*G*) of 237 kJ/mol and is therefore an uphill reaction. Energy input is therefore indispensible for this reaction to proceed. In principle, the photocatalytic reaction over semiconductors is triggered by the direct absorption of a photon by the band gap of semiconductor materials (Figure 1). Upon photon excitation, the photogenerated charges move to the surface of semiconductor particles where photocatalytic reactions occur. Consequently, the efficiency of photocatalytic water splitting is closely affected by the band structure of the semiconductors [8].

The band gap of semiconductor photocatalysts must be larger than the potential of water electrolysis to meet the energetic requirement for overall water splitting (1.23 eV, corresponding to an absorption threshold of 1000 nm). In particular, the bottom level of the conduction band (CB) must be more negative than the reduction potential of water, while the top level of the valance band (VB) should be more positive than the oxidation potential of water. In order to utilize the abundant visible light from the sun, the band gap of photocatalysts has to be less than 3.0 eV (corresponding to an absorption threshold larger than 420 nm). Efficient utilization of these huge amounts of "low energy" photons is crucial to the realization of commercial solar photocatalytic hydrogen production. To this end, band engineering is necessary to design semiconductor photocatalysts with satisfactory hydrogen production efficiency. In addition, the photocatalytic efficiency also depends on the fate of photogenerated hole–electron pairs. To improve the quantum efficiency (QE) of a photocatalytic process, two sequential steps, (1) the efficient separation and transfer of the photogenerated charge carriers to the target surface reaction sites within their life time to avoid their recombination in the form of heat dissipation, and (2) the rapid implementation of reduction and oxidation reactions by those excited charges, should be promoted.

Among the various approaches, nanostructure design with well-tailored band alignment provides a powerful means to the improvement of the photocatalytic activity. In the process of our study, we have also paid special attention on the nanoscale control of the material morphology and construction of functionalized nanostructures to promote charge separation and prolong the lifetime of the photoexcited charge carriers. Our discussion is principally organized around this topic, which constructs the main theme of this review.

### Strategies for the development of various functionalized nanostructures

#### Nanosized functionalized morphology

As mentioned above, absorption of photons with energy equal or higher than the band gap of the semiconductor could lead to photogenerated electrons (e^−^) and holes (h^+^) within conduction and valence bands, respectively. Generally, the photocatalytic reaction often occurs at the semiconductor surface. So the ability to accelerate the migration of photogenerated charges from the interior of the semiconductor to its surface avoiding their bulk recombination enables substantial improvement of photocatalytic efficiency. In this regard, semiconductors with nanosized functionalized morphology could be a beneficial choice. Since the first reports on mesoporous silica MCM-41 [9], inorganic oxides with controlled porosity have been extensively investigated. It is known that mesoporous photocatalysts usually exhibit high specific surface areas that can provide more surface reactive sites. The nanoscale channel walls of mesoporous photocatalysts can also facilitate the migration of photogenerated charges from bulk to surface [10]. It has been reported that porous semiconductor photocatalysts, such as TiO_2_ [11], Nb_2_O_5_ [12] and Ta_2_O_5_ [10], displayed much better photocatalytic properties than their bulk counterparts. Meanwhile, when semiconductor nanocrystals are dispersed in the mesoporous substrate, greatly enhanced photocatalytic properties can also be achieved. For example, dispersing TiO_2_ nanoclusters in MCM-41 [13] and MCM-48 [14] resulted in much higher photocatalytic activities than that of bulk TiO_2_ under UV light irradiation. Our work was carried out on Ni modified mesoporous TiO_2_ photocatalysts [15]. It was found that Ni ions were highly dispersed in the framework of mesoporous TiO_2_ resulting in enhanced hydrogen production in methanol aqueous solution compared to Ni-doped particulate TiO_2_. The photocatalytic activity was found to have a close relationship with the doping amount of Ni ions and the highest activity was obtained when the amount of Ni doping was 1%. Here, the enhanced photocatalytic activity was attributed to doped Ni^2+^ ions which served as shallow trapping sites, preferentially trapping photoexcited holes. The assumption of the role of Ni^2+^ as shallow trapping sites could be rationalized by considering that the energy level of Ni^2+^ is located very close to the valence band edge of TiO_2_ [16]. After trapping a hole, Ni^2+^ will be oxidized to Ni^3+^. However, due to the instability of Ni^3+^, it will quickly return to Ni^2+^ again. The shallow trapping can therefore separate the arrival of photogenerated charges at the surface, so the recombination at the channel surface of mesopores could be greatly reduced. However, when the doping concentration is higher than the optimal level, or for too large particles, the exited hole can be trapped more than once, and recombine with the electron excited by another photon. An optimal dopant concentration is therefore crucial. In another example, we also synthesized Fe-doped mesoporous Ta_2_O_5_ that showed an enhanced activity compared to the bulk counterpart [17].

**Figure 2:**
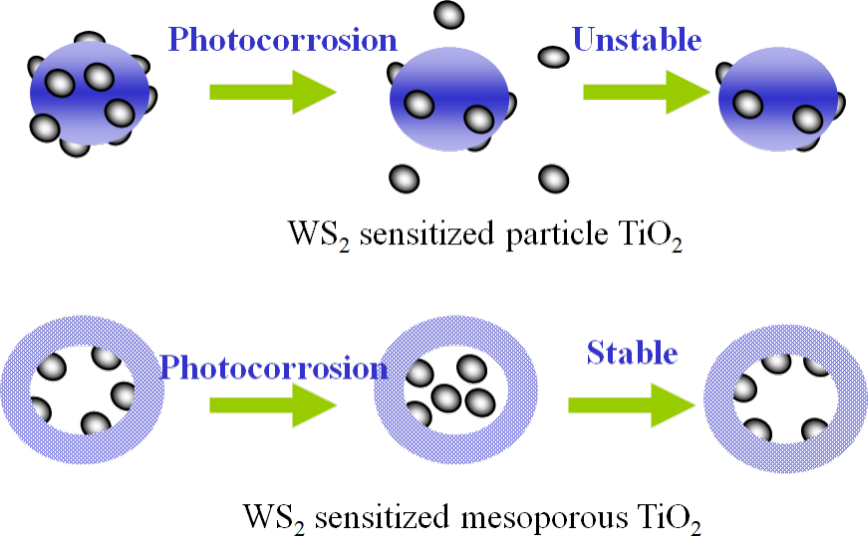
Illustration of mesoporous wall on the anti-photocorrosion of sulfide photocatalyst.

It should be pointed out that TiO_2_ can only respond to UV light, even metal ion doping can hardly enhance its visible light activity. Sensitization with dyes or nanocrystals is one possible approach to extend the light absorption of TiO_2_. Sreethawong et al. found that eosin Y-sensitized mesoporous-assembled Pt/TiO_2_ nanocrystal photocatalysts exhibited enhanced photocatalytic hydrogen production under visible light irradiation [18]. Lee et al. also reported that CdS and CdSe nanocrystals dispersed on the internal surface of mesoporous TiO_2_ films could lead to the promoted photocatalytic hydrogen production under visible light [19]. We have also investigated the visible-light-driven photocatalytic performance over a nanosized WS_2_-sensitized mesoporous TiO_2_ photocatalyst [20]. Compared to bulk TiO_2_ without mesopores, more WS_2_ can be loaded in the mesoporous TiO_2_. Moreover, the mesoporous channels can prevent the light-induced detachment of WS_2_ nanoparticles from the substrate during the photocatalytic reaction. These factors resulted in much higher activity and better stability, as schematically illustrated in Figure 2. Here the elevation of the conduction band of nanosized WS_2_ due to quantum confinement effect is considered to be crucial. As the recombination of photogenerated charges within TiO_2_ could be neglected, the rate-determining step for photocatalytic reaction is the electron transfer from TiO_2_ to H^+^ in the solution. Therefore, deposition of Pt as cocatalyst is indispensable for an efficient hydrogen evolution.

**Figure 3:**
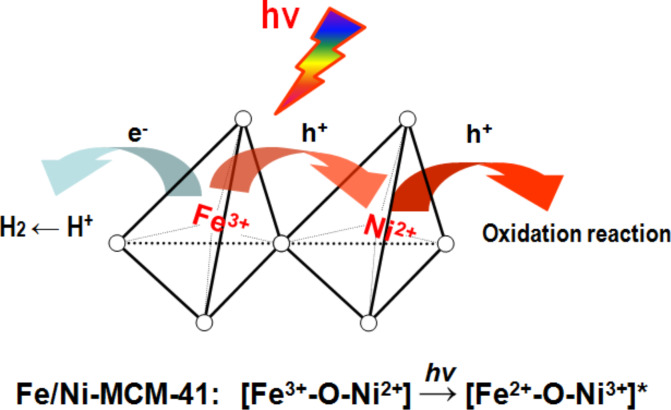
Proposed mechanism for charge transfer in Fe/Ni co-doped MCM-41. Reprinted with permission from [21]. Copyright (2014) Elsevier.

Generally, MCM-41 is not photo-reactive. But it can be activated by coupling with a semiconductor or doping a transitional metal. Figure 3 shows the proposed charge separation mechanism within a representative transitional metal-containing molecular sieve photocatalyst [21]. In principle, after excitation by visible light, photogenerated electrons are transferred from CdS to the Ti-MCM-41 substrate to conduct a reduction reaction. The holes, on the contrary, remain at the CdS to let oxidation reactions occur. We also investigated the effect taken by transitional metal doping such as Cr or/and Ti incorporation in MCM-41. Active visible light absorption sites could be generated in MCM-41 due to transitional metal doping. It was found that the highly dispersed Cr ions within MCM 41 could be easily excited by visible light irradiation due to the electron transfer from O^2−^ to Cr^6+^. Such excited states are active for charge transfer and thus showed a relatively high photocatalytic activity. Accordingly, the unique arrangement of the localized charges in the ordered mesoporous structure leads to a significantly prolonged life time of electron–hole pairs compared to traditional metal ion doped semiconductors.

As was found, Cr-Ti-MCM-41 showed much higher activity than Cr-MCM-41 with similar amount of Cr doping for two kinds of MCM-41 material. Due to the presence of Ti, the photocatalytic mechanism of Cr-Ti-MCM-41 is quite different from that of Cr-MCM-41. For the former material, the Cr–O–Ti interaction should be responsible [22]. In this case, a second metal element introduced as a donor can closely link to the first metal element by forming an oxo bridge, which is supposed to be robust chromophore induced by metal-to-metal charge-transfer (MMCT).

#### Nanosized functionalized surface

A noble-metal cocatalyst, such as Pt, is usually indispensable for many photocatalysts to achieve high photocatalytic activities for hydrogen production. It was recently reported that the valance state of platinum plays an important role in the hydrogen production efficiency, and oxidized platinum was demonstrated to be more efficient than metallic platinum as cocatalyst for hydrogen production [23–24]. Taking into account the cost of the designed photocatalyst for commercial purposes, the development of noble-metal free cocatalysts is still valued. Alternative cocatalysts such as MoS_2_ have been reported to be effective to enhance photocatalytic H_2_ evolution on CdS [25]. Low-cost WC was also used as efficient cocatalyst on CdS because of its low overpotential for hydrogen production and proper physicochemical properties [26]. The Xu group has studied the effect of NiS working as a cocatalyst. A simple hydrothermal method was used to synthesize NiS/CdS photocatalysts, which have a remarkably high QE of 51.3% at 420 nm in lactic acid sacrificial solution [27]. Co-loading of both reduction and oxidation cocatalysts on the semiconductor was also suggested to be able to enhance the photocatalytic hydrogen production [28].

**Figure 4:**
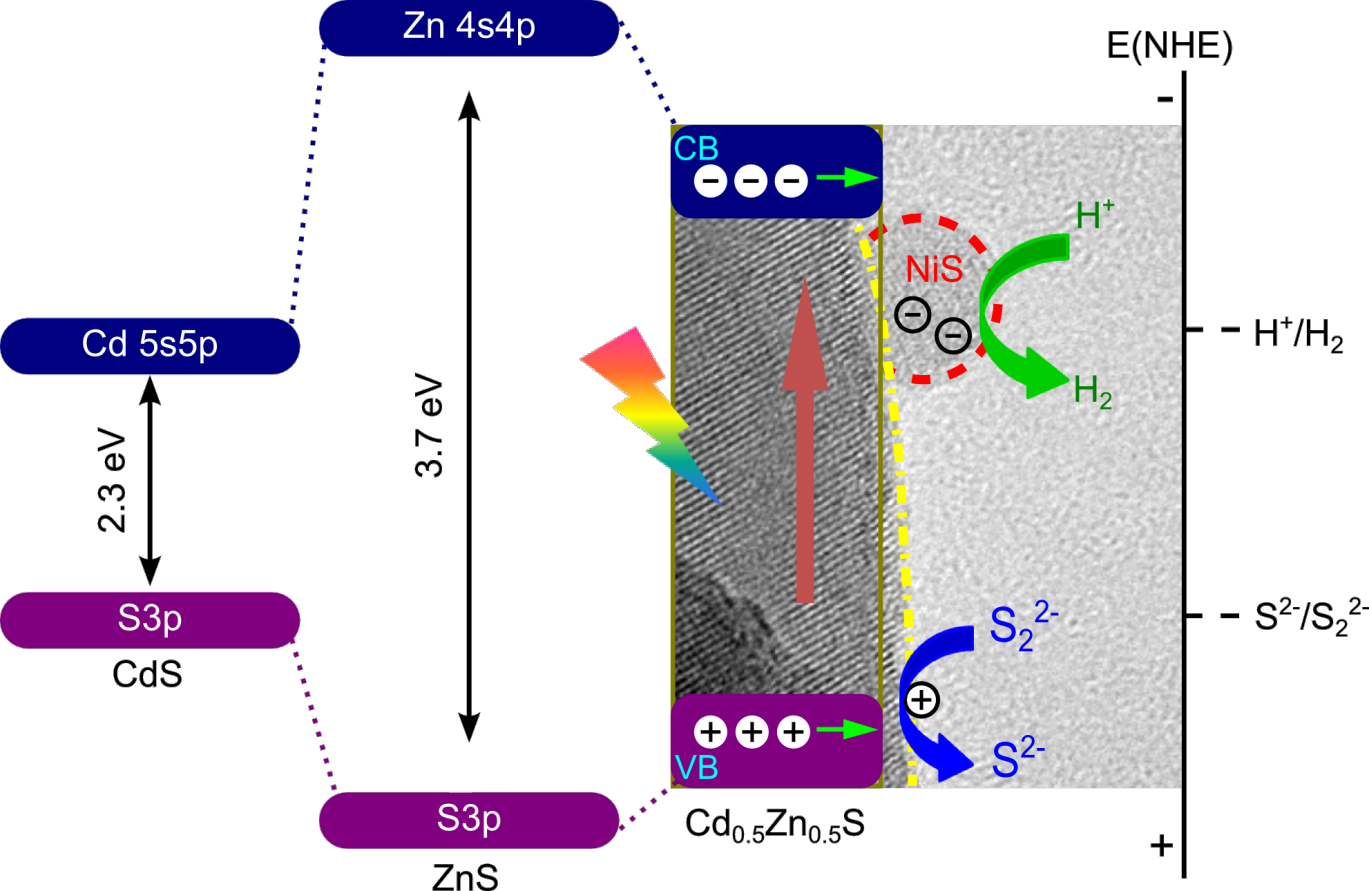
Proposed photocatalytic charge separation process over the band-structure controlled NiS/Cd_0.5_Zn_0.5_S photocatalyst. Reprinted from with permission from [29]. Copyright (2010) Elsevier.

Here, it is assumed that the photocatalytic activity could be significantly improved if the cocatalysts were loaded especially on a nanosized functionalized surface. We reported the design and preparation of a highly efficient Cd_0.5_Zn_0.5_S photocatalyst decorated with nanosized NiS surface heterojunctions. The hydrogen evolution rate over this photocatalyst could reach 1.4 mmol/h, with a QE of 33.9%. This efficiency is much higher than that of many photocatalysts containing noble metals. As shown in Figure 4, in the hybrid photocatalyst, the NiS nanoparticles can serve as electron trapping sites and extract photogenerated electrons from Cd_0.5_Zn_0.5_S substrate, which finally leads to spatially separated photoreduction and oxidation reactions. NiS plays a similar role as a noble metal, which can provide active sites for proton reduction, and thus efficiently enhance the overall photocatalytic activity. Our work demonstrates that efficient and low cost photocatalytic hydrogen production can be achieved through the substitution of noble metal cocatalyst with a properly engineered surface heterojunction [29].

In addition to the screening for a noble-metal free cocatalyst, we have also tried to modify the surface of the photocatalyst, aiming to form functionalized surface nanostructures. A highly active and stable CdS photocatalyst was obtained by a two-step thermal sulfuration method. Nanostep structures formed at the surface of the CdS photocatalyst, leading to a significantly improved photocatalytic activity compared to CdS prepared by traditional approaches [30]. The enrichment of Pt around nanostep regions revealed the preferred migration of photoexcited electrons there and the reduction H_2_PtCl_6_ precursor to metallic Pt. Similar nanosteps were also reported by Kudo for another metal sulfide photocatalyst [31]. Although the mechanism for the formation of nanostep structures could be different, the important role of the nanostep surface for the enhancement of photocatalytic performance was also shown.

**Figure 5:**
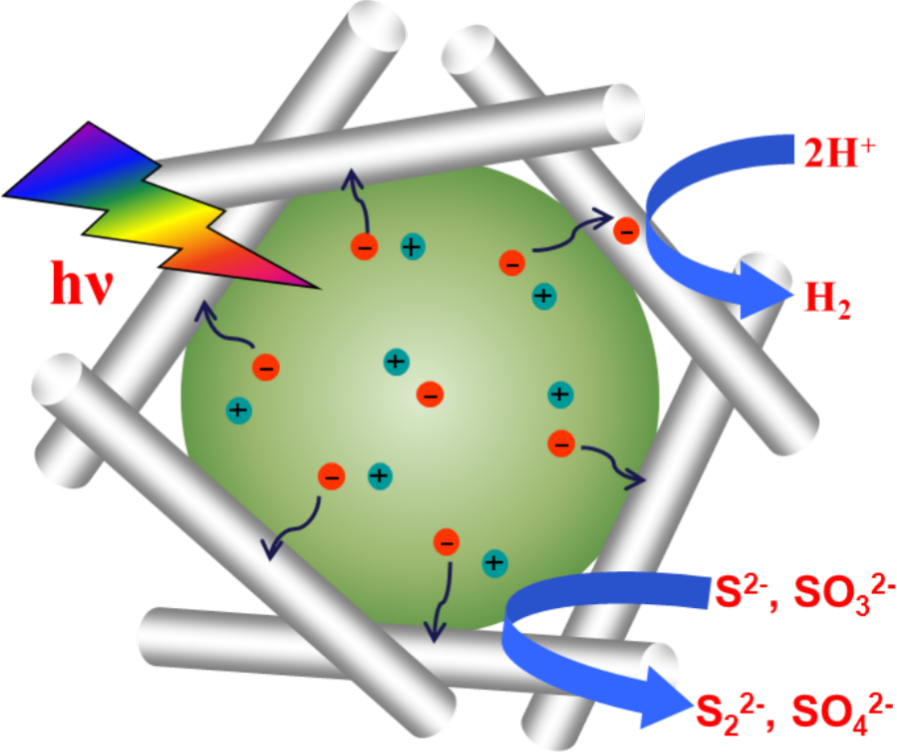
CdS nanoparticle enwrapped by the surrounding TNTs showed a significant enhancement of charge separation and therefore a high activity. Reproduced with permission from [33]. Copyright (2011) The Royal Society of Chemistry.

The surface architecture of composite photocatalysts can significantly affect the photocatalytic process. For example, Jang and co-workers reported that bulky CdS decorated with TiO_2_ nanoparticles was much more active than bulky TiO_2_ decorated with CdS nanoparticles [32]. We have prepared CdS/titanate nanotubes (CdS/TNTs) photocatalysts with a unique morphology by a simple one-step hydrothermal method [33]. As schematically illustrated in Figure 5, the CdS nanoparticle was intimately enwrapped by the TNTs, resulting in a remarkably enhanced charge separation efficiency and thereby photocatalytic hydrogen production activity. The similar enwrapped structure can also be achieved for Cd_0.5_Zn_0.5_S/TNTs nanocomposites [34]. The QE at 420 nm over the nanocomposites reached 38.1% without loading any cocatalyst. Meanwhile, except for the greatly reduced toxicity by using Zn^2+^ instead of Cd^2+^ (Cd content, 4.0 wt %), Cd_0.5_Zn_0.5_S/TNTs also showed a good stability for hydrogen production. These factors are significantly beneficial for their further application in the field of solar energy conversion.

#### Crystal facets engineering

As we know, the surface of a given semiconductor nanocrystal usually consists of certain crystal facets that occupy specific coordination numbers and atom ratios, leading to a deviation of their band structure from their bulk counterpart. Consequently, some crystal facets have shown higher activity than other facets for many photocatalyst during a certain photocatalytic reaction [35–37]. Jang et al. found that ZnO nanoplates with a preferential exposure of Zn(0001) faces showed high photocatalytic activity [38]. Wang et al. constructed ZnO–CdS heterostructures by employing ZnO rods enclosed with well-developed {1100} and {0001} facets to promote photocatalytic hydrogen evolution [39]. Pan et al. found that {001}, {101} and {010} facets of anatase TiO_2_ had different photocatalytic activities. However, when partially terminated with fluorine, the three facets had similar photocatalytic activity in H_2_ evolution [40]. Engineering the morphology of semiconductors to preferably expose active facets is therefore a promising approach, yet a big challenge to date. Selecting Cu_2_WS_4_ as model photocatalyst, we obtained interesting decahedral morphologies by a one-step hydrothermal method. The hydrothermal method avoids the using of toxic H_2_S gas and simplifies the catalyst preparation process. Owing to the oriented growth and the presence of a large percentage of active (001) crystal planes, the photocatalyst showed high visible-light activity. The highest activity was obtained over the Cu_2_WS_4_ decahedrons that were hydrothermally prepared at 200 °C for 72 h, with an QE at 425 nm for photocatalytic hydrogen production of 11% [41].

**Figure 6:**
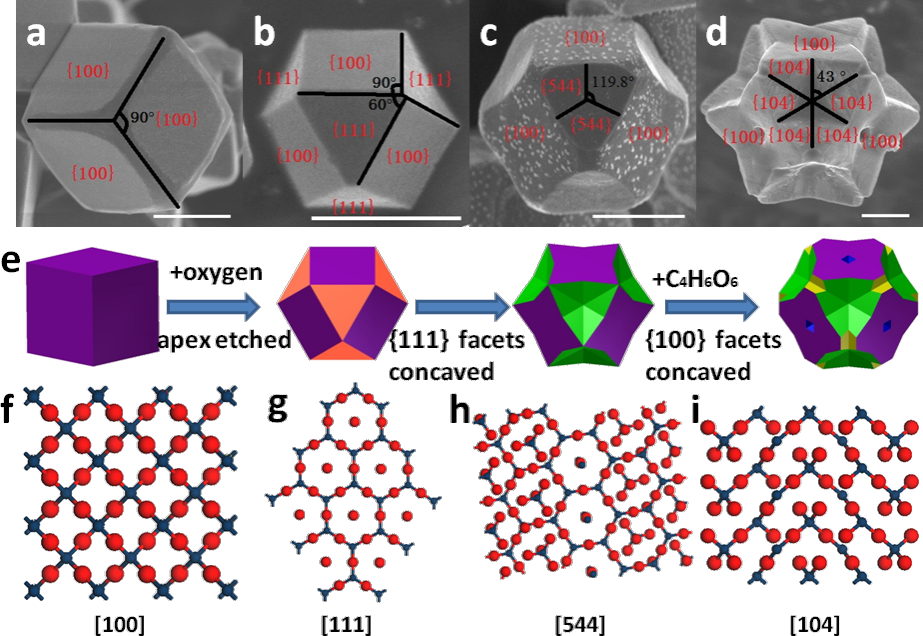
Morphological evolution of Cu_2_O prepared with different reaction times, (a) 30 min, (b) 60 min, (c) 90 min and (d) 120 min, all scale bars are 1 μm. Reprinted with permission from [45]. Copyright (2014) The Royal Society of Chemistry.

Regarding the stability, oxide photocatalysts would be better than sulfide and nitride photocatalysts [42]. Regrettably, oxide semiconductors frequently possess either low conduction band positions or wide band gaps, which seriously impair their suitability as photocatalysts for hydrogen production [43]. Among the very few visible-light-responsive photocatalysts, cuprous oxide (Cu_2_O) deserves our special attention. Cu_2_O is a p-type oxide semiconductor with a direct band gap of 2.0 eV. Theoretically, its light-to-hydrogen conversion efficiency can reach 18% [44]. We have thus devoted to the fabrication of Cu_2_O nanocrystals with controlled shape to improve its photocatalytic activity for hydrogen production. As shown in Figure 6, multifaceted Cu_2_O with controlled crystal facets exposure has been prepared through a facile one-step method. It was revealed that photogenerated electrons preferred to accumulate on high-indexed facets, while photogenerated holes tended to migrate to {100} facets, leading to an efficient spatial charge separation and thereby enhanced photocatalytic hydrogen production from reforming of glucose over the Cu_2_O polyhedron [45]. The origin of the charge separation on different crystal facets of TiO_2_ has been already theoretically calculated [40]. A slight band offset was observed in both valance and conduction band between two different facets, driving the charge transfer from one facet to another. Our research indicated that a controlled fabrication of different crystal facets with separated functions, such as separated oxidation and reduction sites is one of the effective approaches to the enhancement of the activity of the existing semiconductors.

**Figure 7:**
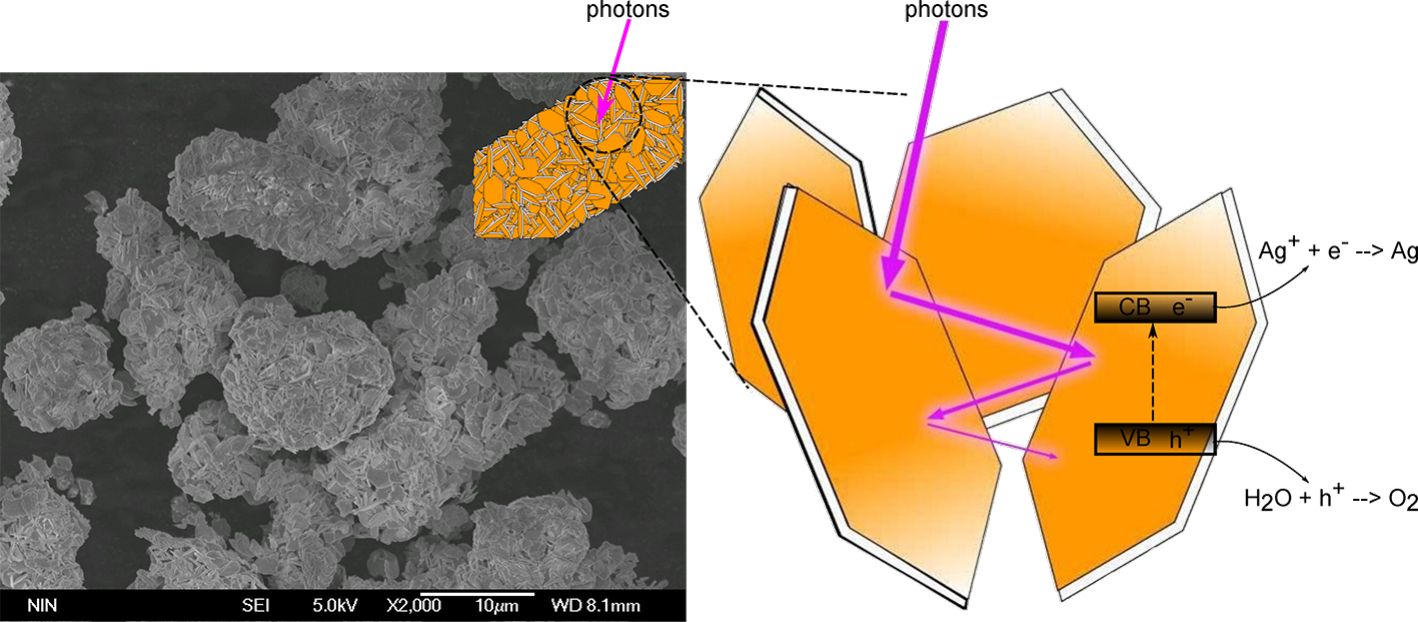
Enhanced light absorption ascribed to multiple light reflections in the nanosheet-based hierarchical structure.

Perovskite ABO_3_ is another group of metal oxide materials deserving in-depth exploring. Most of metal elements can be stably located in ABO_3_ structures, and functionalized multicomponent ABO_3_ materials can thus be prepared by partial substitution of cations in A and B sites [46–48]. In this approach, we have successfully synthesized hexagonal single-crystal nanosheet-based NaSbO_3_ and AgSbO_3_ hierarchical cuboidal microclusters with exposed {001} facets via a facile and surfactant-free hydrothermal reaction. The light absorption, charge separation and surface reaction were simultaneously optimized through the unique structure assembled from nanosheets, leading to the greatly enhanced photocatalytic activity [49]. Micro- or nanoscale surface-structuring can increase the degree of horizontal light distribution via light scattering. Otherwise, the trapped photons would be lost by direct reflection from a flat surface [50]. As shown in Figure 7, enhanced light absorption arising from multiple light reflections in the nanosheet-based hierarchical structure could be achieved over NaSbO_3_ and AgSbO_3_ photocatalysts. Meanwhile, the single-crystal nature can reduce the crystal defects leading to more efficient charge separation. The larger surface area provides more active sites for photocatalytic reaction. The exposed {001} facets as the reactive surfaces can accelerate the redox reactions. Therefore, nanosheet-based AgSbO_3_ photocatalysts showed a 1.8 times higher initial O_2_ evolution rate than AgSbO_3_ photocatalysts without the hierarchical structure under visible-light irradiation.

Besides facet control, microstructure refinements of the crystal facets, such as distortion of the lattice and configuration of certain crystal facets, can also promote the charge separation and facilitate charge transfer. For example, we have investigated the effects of synthesis conditions on the structure and the photocatalytic property of ZnIn_2_S_4_ [51–53]. It was discovered that the distance of *d*(001) ZnIn_2_S_4_ could be adjusted by controlling preparation condition. The enlarged *d*(001) spacing led to distorted [ZnS_4_] and [InS_4_] tetrahedrons within the ZnIn_2_S_4_ crystal lattice, which in turn, generated an electrostatic field induced by a change of the dipole moments of the lattice. Such a built-in electrostatic field is obviously favorable for an efficient charge separation and hence leads to enhanced photocatalytic activity.

#### Combined control of band structure and morphology

In principle, a semiconductor photocatalyst should meet at least two requirements to achieve high visible-light photocatalytic activity. A high conduction band is necessary to ensure enough potential for proton reduction. It should also have a narrow band gap in order to utilize incident light to the largest extent. Cd_1−_*_x_*Zn*_x_*S, as the solid solution of CdS and ZnS, has received lots of attention in recent years, owing to the tunable band structure and excellent photocatalytic properties for hydrogen production under visible light without needing noble metal cocatalysts [54]. However, the band gap of Cd_1−_*_x_*Zn*_x_*S is not narrow enough for an efficient utilization of visible light. As reported by Kudo et al, a donor level above the valence band of ZnS could be formed when it is doped with Ni^2+^, which is responsible for the visible light response of Ni-doped ZnS [55]. It inspired us to further tune the band structure of Cd_1−_*_x_*Zn*_x_*S solid solution by Ni doping. Ni^2+^-doped Cd_1−_*_x_*Zn*_x_*S microspheres were prepared in our work. Here, the doped Ni^2+^ is expected to form a donor level above the valence band of Cd_1−_*_x_*Zn*_x_*S and increase its visible light absorption. At the same time, its high conduction band can be still maintained. The enhanced photocatalytic activity was thus achieved [56]. Moreover, Cd_1−_*_x_*Zn*_x_*S could be also modified by doping with alkali metals. We have also successfully synthesized Sr and Ba doped Cd_1−_*_x_*Zn*_x_*S solid solution photocatalysts with improved activity [57–58]. The underlying role of the doping ions might be quite different. However, the band structure of the solid solution was similarly affected.

**Figure 8:**
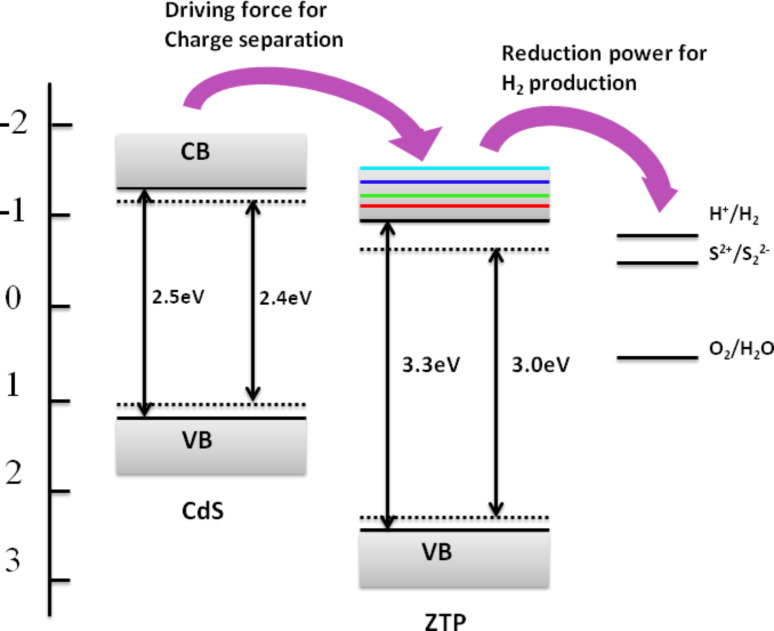
Schematic illustration for the charge separation in CdS/mesoporous ZTP. Reprinted with permission from [60]. Copyright (2007) The American Chemical Society.

Mesoporous zirconium–titanium mixed phosphates (ZTP) is also a photocatalyst of interest as they not only show both cation- and anion-exchange capacity, but also can split pure water for hydrogen production under UV light irradiation [59]. These properties make ZTP an ideal candidate after coupling with CdS to form visible-light composite photocatalysts. In addition, due to the adjustable Zr to Ti ratio, the optical properties of this material can be readily tuned. Employing ZTP as substrate and functional constituent, a novel CdS/mesoporous ZTP composite photocatalyst was successfully synthesized via the two-step thermal sulfuration method. We have found that the prepared composite photocatalyst displayed superior activity compared to that prepared by direct sulfuration at room or high temperatures. As shown in Figure 8, the conduction band of ZTP could be continuously controlled by regulating the Zr/Ti ratio. At the optimal Ti to Zr ratio of 3, the energy difference between conduction bands of CdS and ZTP could ensure a large driving force for fluent electron transfer from CdS to ZTP, while the electron localized on the ZTP substrate is still sufficiently active for hydrogen evolution. Combination of CdS with band adjustable mesoporous ZTP could thus inherit the advantage of ZTP in terms of both morphology and band structure. The QE of this composite photocatalyst at 420 nm was determined to be 27.2% [60].

**Figure 9:**
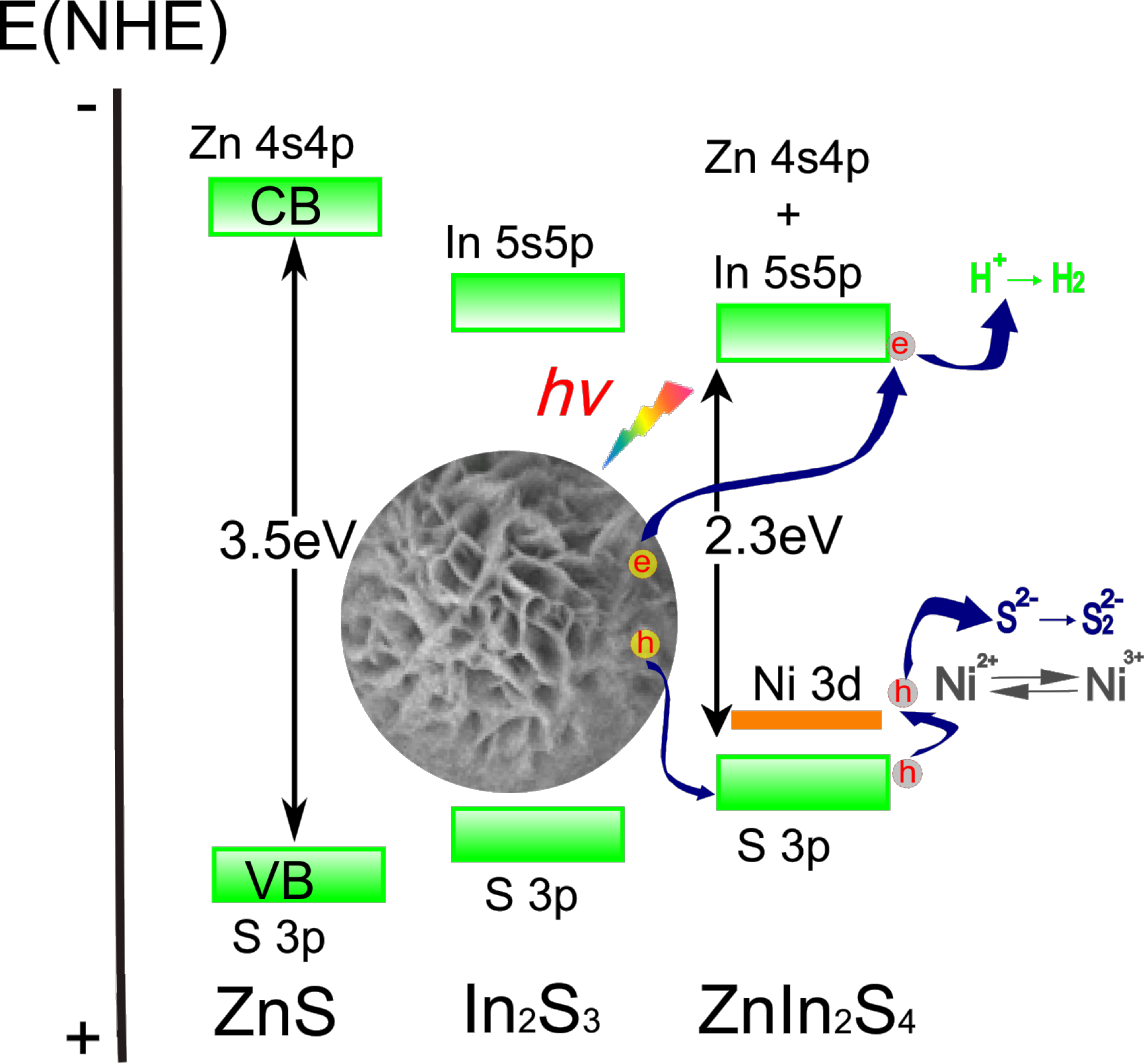
Photocatalytic mechanism over Ni-doped ZnIn_2_S_4_ with plenty of curved nanosheets. Reprinted with permission from [61]. Copyright (2010), Springer.

In addition, we also prepared flower like Ni-doped ZnIn_2_S_4_ with plenty of curved nanosheets using hydrothermal method, as shown in Figure 9. It was found that there existed an optimal Ni doping concentration, i.e., 0.3 wt %, for the highest hydrogen production efficiency. Only Ni ions occupying crystal lattice sites of ZnIn_2_S_4_ were found to be active. A higher Ni doping is not effective due to the limited solubility of Ni in the lattice of ZnIn_2_S_4_ and could be detrimental owing to the coverage of surface reaction sites. The existence of thin nanoscale sheets is regarded to be important for its increased activity. Ni^2+^ ions were also expected to be shallow trapping sites, which could enhance the charge separation [61]. Similar combined control of band structure and morphology were also carried out by other groups. Kudo and co-workers prepared layered AGa_2_In_3_S_8_ (A = Cu or Ag) by a solid state method for photocatalytic hydrogen production. Both CuGa_2_In_3_S_8_ (1.91 eV) and AgGa_2_In_3_S_8_ (2.27 eV) showed a quite high photocatalytic activity [62]. Chen and co-workers synthesized hierarchical ZnS–In_2_S_3_–CuS nanospheres with a nanoporous structure. A high QE of 22.6% at 420 nm is achieved without loading cocatalysts due to their high crystallinity, high surface area and unique microstructure [63].

As the crystal size of the semiconductor is close to the exciton Bohr radius, its bandgap can be enlarged with a reduced crystal size due to the quantum confinement effect. Therefore, we have synthesized Co_3_O_4_ quantum dots (3–4 nm) via a facile reverse micelle method for the first time. Co_3_O_4_ has been widely employed as a photocatalyst for oxygen production due to its nontoxicity, low cost and narrow band gap (2.1 eV). However, photocatalytic hydrogen production could not be achieved, because the conduction band edge of bulk Co_3_O_4_ is not negative enough to reduce H^+^. Compared with bulk Co_3_O_4_, Co_3_O_4_ quantum dots have a wider bandgap. Valence-band XPS spectra showed that the valance band maxima (VBM) of Co_3_O_4_ quantum dots and bulk Co_3_O_4_ are almost at the same position, implying that the enlarged bandgap of Co_3_O_4_ quantum dots was mainly ascribed to the conduction band minimum (CBM) upshift. Due to the negative shift of the conduction band, Co_3_O_4_ quantum dots can split pure water into O_2_ and H_2_ stoichiometrically under visible light irradiation without any cocatalyst. This is the first report to date of Co_3_O_4_ photocatalysts capable of splitting pure water, which provides a new route to the development of nanosized photocatalysts for water splitting [64].

#### From single heterojunction to long range ordered homojunction

Semiconductor heterostructures can be engineered to combine functionalities that result from the bulk properties of their constituent phases with properties that are directly related to the electronic and atomic character of their interfaces [65]. The concept has been proven in photovoltaic cells and optoelectronic devices, where junction-type semiconductors show greatly improved efficiency compared with junction-free semiconductors [66]. Depending on the band gaps and the electronic affinity of semiconductors, semiconductor heterostructures can be divided into three different cases: type-I, type-II and type-III band alignment [67]. In a type-II band alignment, the band offsets in the conduction and valance bands go in the same direction, resulting in a band bending at the interface between two semiconductors. Such band bending consequently induces a built-in field, which drives the vectorial migration of the photogenerated charges at the interface, leading to a spatial separation of e^−^ and h^+^ on different sides of the heterojunction. Most type-II heterojunctions have been obtained from two different semiconductors [68–69]. Matching of two semiconductor materials with both their band positions and crystal lattices is the key challenge of this strategy. Most recently, successful efforts have been made to fabricate heterojunctions of different phases of the same material. Bao and co-workers prepared CdS photocatalysts with different phases for photocatalytic hydrogen production [70]. Interestingly, a higher photocatalytic activity is observed from the composite of hexagonal and cubic CdS as compared to single hexagonal or cubic CdS. However, the relation of electronic interaction between different phases with the photocatalytic activities was not discussed in their study [70]. Li and co-workers have demonstrated the greatly enhanced photocatalytic overall water splitting over an α–β phase junction of Ga_2_O_3_ [71]. The improved photocatalytic activity results from the efficient charge separation and transfer across the α–β phase junctions of the Ga_2_O_3_ particles.

**Figure 10:**
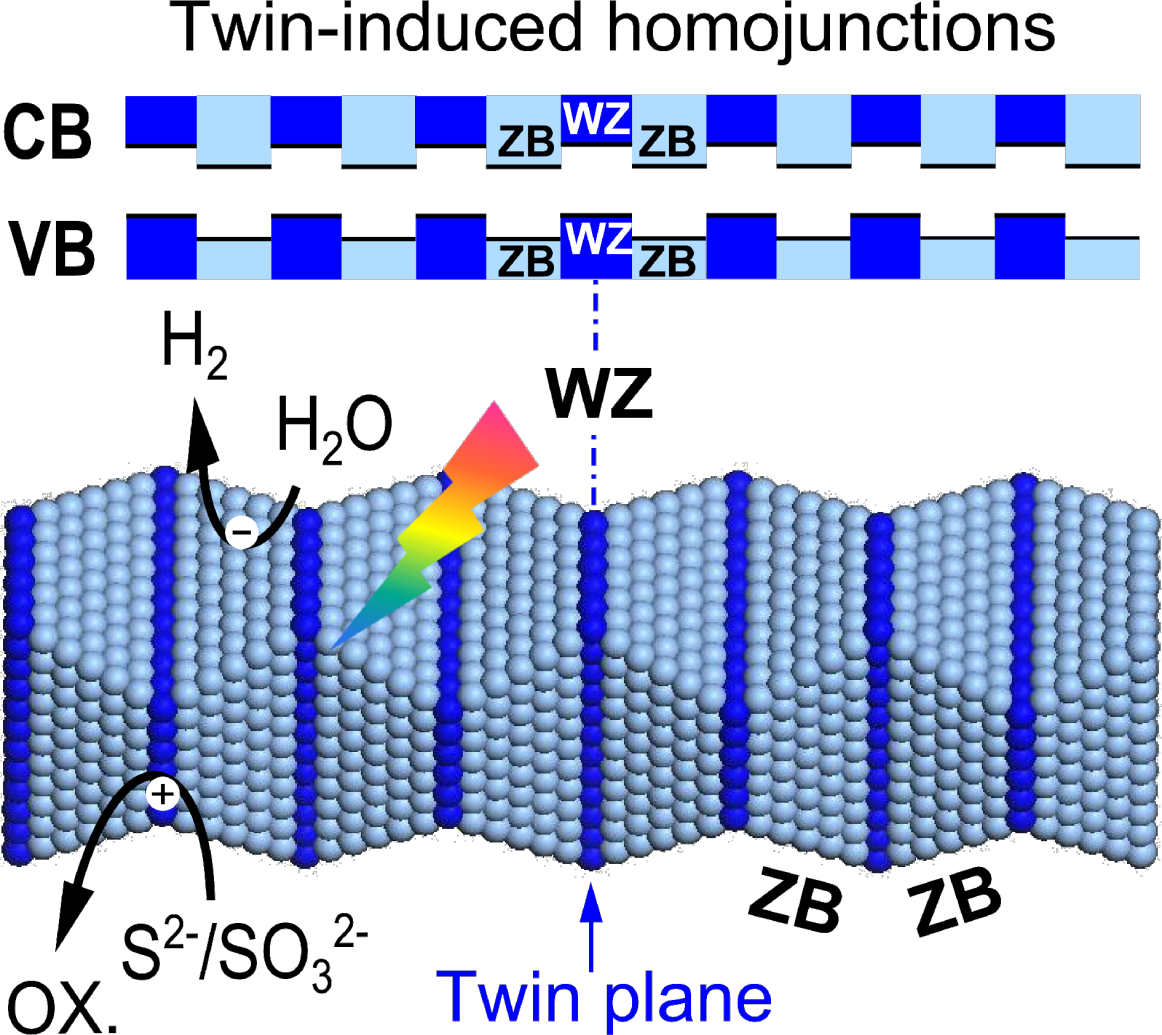
Illustration of twin-induced 1D long range ordered homojunctions over Cd_0.5_Zn_0.5_S with alternative zinc-blende (ZB) and wurtzite (WZ) segments.

We have found that spherical twin-containing noble-metal-free Cd_0.5_Zn_0.5_S is a superb photocatalyst for hydrogen production, showing a QE of 43% at 425 nm [72]. However, the low density of twin planes and the insufficient control of the crystal shape in these catalysts inspired us to further improve their photocatalytic efficiency by fabricating more effective junctions. Recently, we reported a twinned Cd_0.5_Zn_0.5_S anisotropic nanocrystal with controllable aspect ratios and a high percentage of long-range ordered twin planes. As shown in Figure 10, the densely distributed rotational twin planes are found to be parallel to each other and perpendicular to the <111> direction. More interestingly, zinc-blende (ZB) and wurtzite (WZ) segments alternatively occur along the <111> direction of the designed material, resulting in its unique optical and electrical properties. It was also shown that type-II staggered band alignment between WZ and ZB segments resulted in an immense number of homojunctions in a specific dimension. Unlike the well-known heterojunctions, no foreign-atom doping or combination was required for the formation of these junctions, resulting in the homogeneity of the materials themselves and thereby enabling us to engineer the necessary semiconductor band structures more exactly. Notably, it was possible to raise the QE for the photocatalytic hydrogen formation to 62% because of the improved efficiency in charge separation [73]. These dense homojunctions are clearly superior to the single homojunction formed by introduction of a thin p-type layer on n-type α-Fe_2_O_3_ and creating a built-in field as reported in [74]. We suggest that our results have the potential to lead to a new general method for the synthesis of new, highly active photocatalysts by the application of our method to other semiconducting materials.

## Conclusion

With the rapid development of creative nanomaterials for new energy applications [75], the nanostructures have also been found essential for achieving high efficient photocatalysts and cocatalysts. The heart of the study is the nanostructure design from functionalized nanosized morphology and surfaces of relatively larger scale to the precise tuning of crystal facets and junctions in much smaller scale. In the process of our study, we firstly attempted the simple combination of two or more semiconductors both physically and chemically. Parallel to this advancement, nanosized functionalized morphology and surfaces are employed in these hetero-combinations to obtain enhanced charge generation and separation. We then elaborate on the combined control of band structure and morphology to reveal the synergistic effects by coupling two or three kinds of modifications in one semiconductor. In this case, enhanced matching of two components in one hybrid photocatalyst, in terms of morphologic contact, band structure and crystal lattices could be achieved. Especially, various heterostructure architectures with combined functionality of the constituent phases are suggested to be promising to address the primary requirements for an efficient photocatalytic generation of hydrogen by using solar energy: visible-light activity, chemical stability, appropriate band-edge characteristics, and potential for low-cost fabrication. Finally, we highlight the heterostructure design at a precise nanoscale control, such as materials of same composition but different phases and/or from heterojunction to homojunction engineering. As has been demonstrated by our twinned Cd_0.5_Zn_0.5_S, an ordered homojunction is much effective than a disordered one. Co-existence of two phases in same semiconducting material in an ordered way, preferably with type-II band alignment, is therefore recommended to be the most desired nanostructures enabling photo-chemistry or -electricity conversion. How to construct such nanostructures in a low-cost and time-efficient way is the challenge ahead.
